# Supplementary figures and images for: A previously unrecognized superfamily of macro-conotoxins includes an inhibitor of the sensory neuron calcium channel Cav2.3
Source: PLoS Biol. 2023 Aug 3;21(8):e3002217. doi: 10.1371/journal.pbio.3002217 (PMC10437998; doi:10.1371/journal.pbio.3002217)

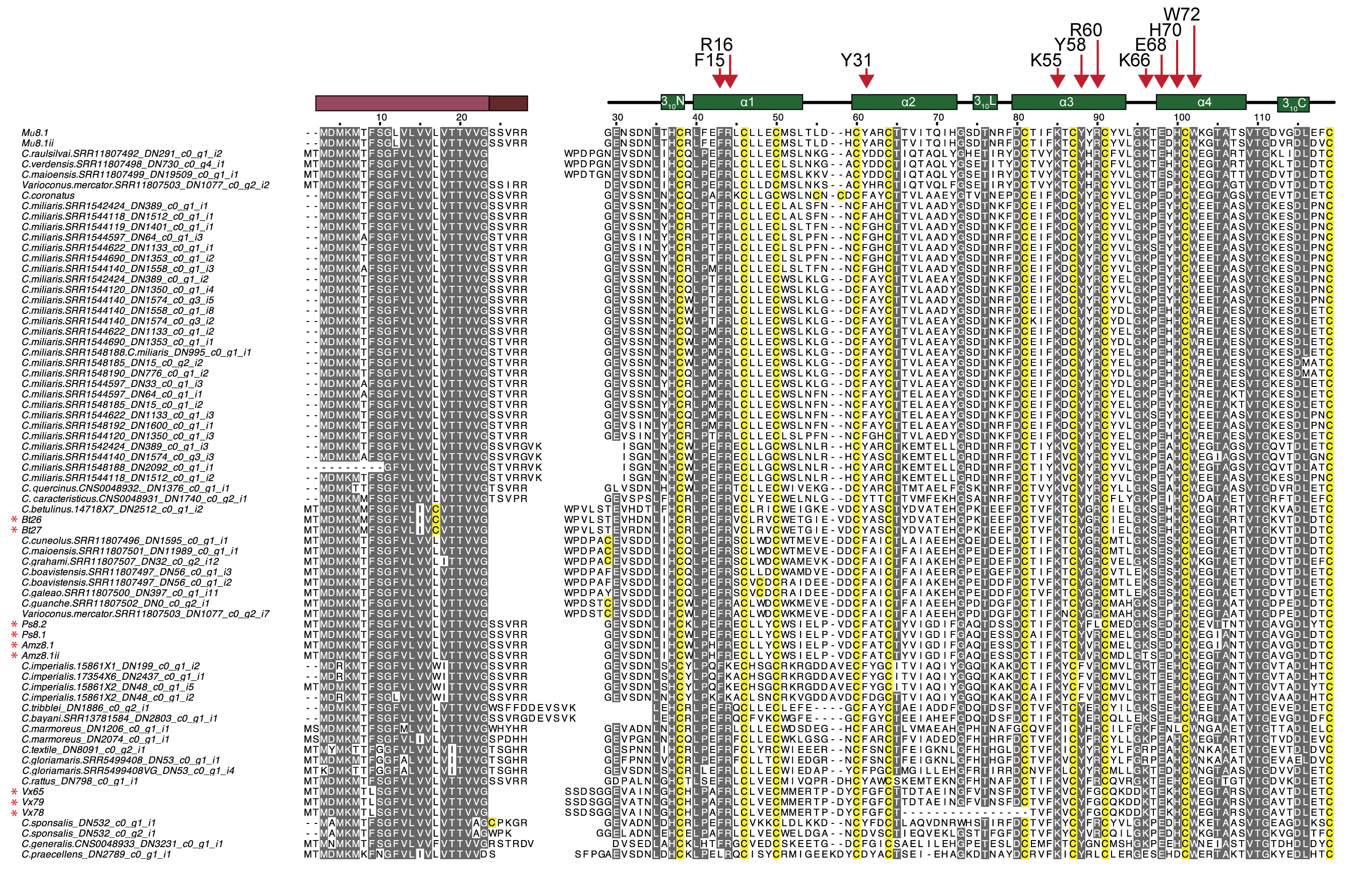

Supplement: S1 Fig — Sequences harvested from venom gland transcriptomes available in the NCBI, DDBJ, and CNGB repositories using the precursor sequence of Mu8.1 as query. Truncated sequences, duplicates, and variants with only 1–2 amino acid residue differences were not included. The remaining sequences were used in a multiple sequence alignment carried out using the MAFFT version 7 multiple alignment online interface [61] and visualized in Jalview [62]. For clarity, the signal and propeptide sequences are depicted with a space preceding the mature toxin sequences. Amino acid residues are shaded in gray according to a 90% identity threshold (all cysteine residues are shaded yellow regardless of conservation). Prepro-sequences are annotated with colored bars (positioned according to the Mu8.1 sequence) indicating the tripartite organization. Mauve: signal sequence; maroon: propeptide region; green: mature conotoxin, here drawn to illustrate the α-helical structure as it corresponds to the Mu8.1 sequence. Red asterisks indicate those sequences previously annotated as con-ikot-ikot uncovered by pBLAST searching as described in the main text. Red arrows highlight amino acid residues referred to throughout the main text, and green rectangles represent α-helices. (TIF) [file pbio.3002217.s001.tif]

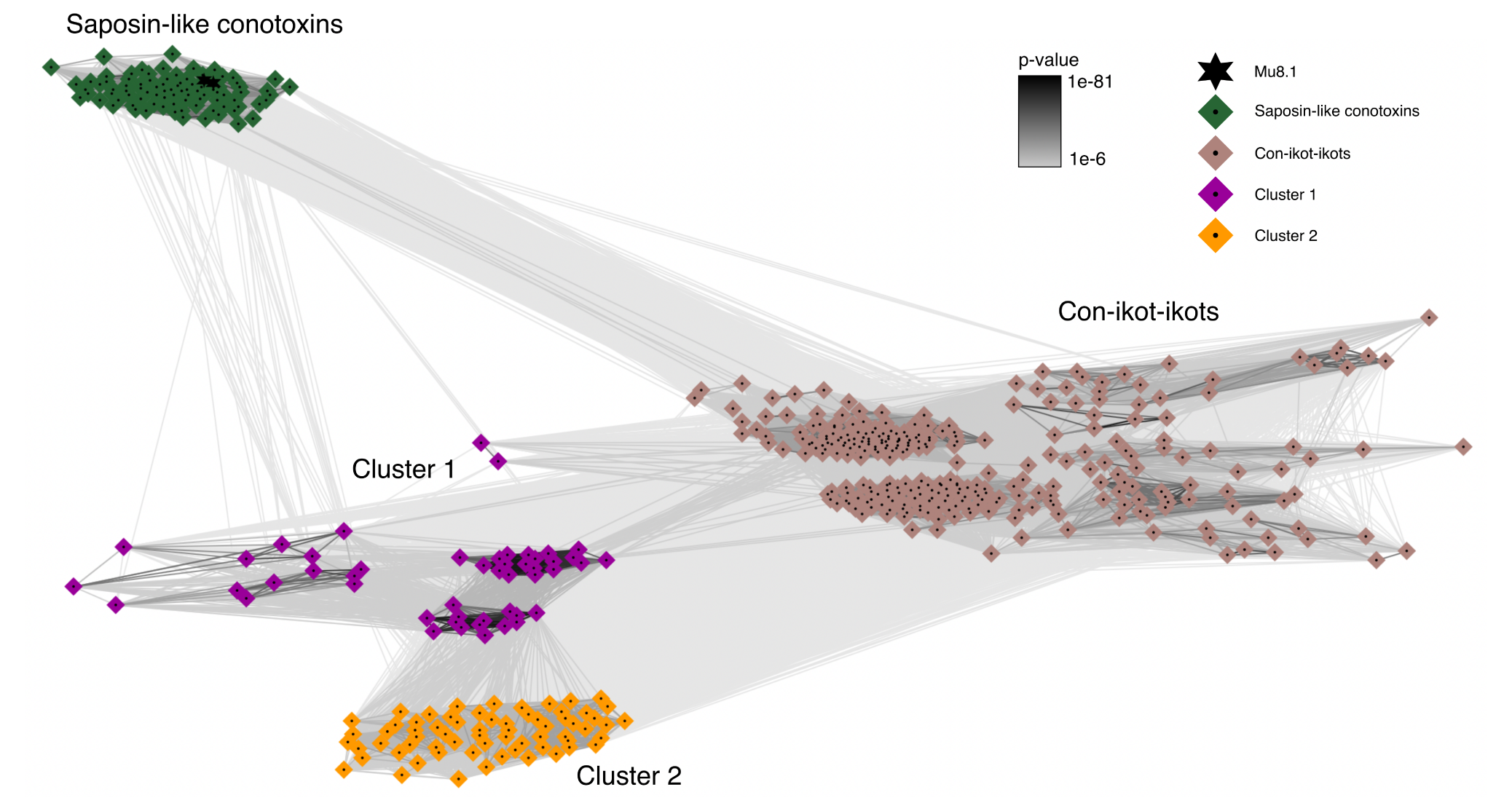

Supplement: S2 Fig — The nodes depict individual precursor amino acid sequences, and the edges correspond to the BLAST p-values < 1 × 10^−6^ between the nodes. The 4 clusters are labelled and color-coded according to the legend in the upper, right-hand corner. Mu8.1 and Mu8.1ii are furthermore highlighted by the star shapes. Processed data for cluster analysis found in S1 Data. (TIF) [file pbio.3002217.s002.tif]

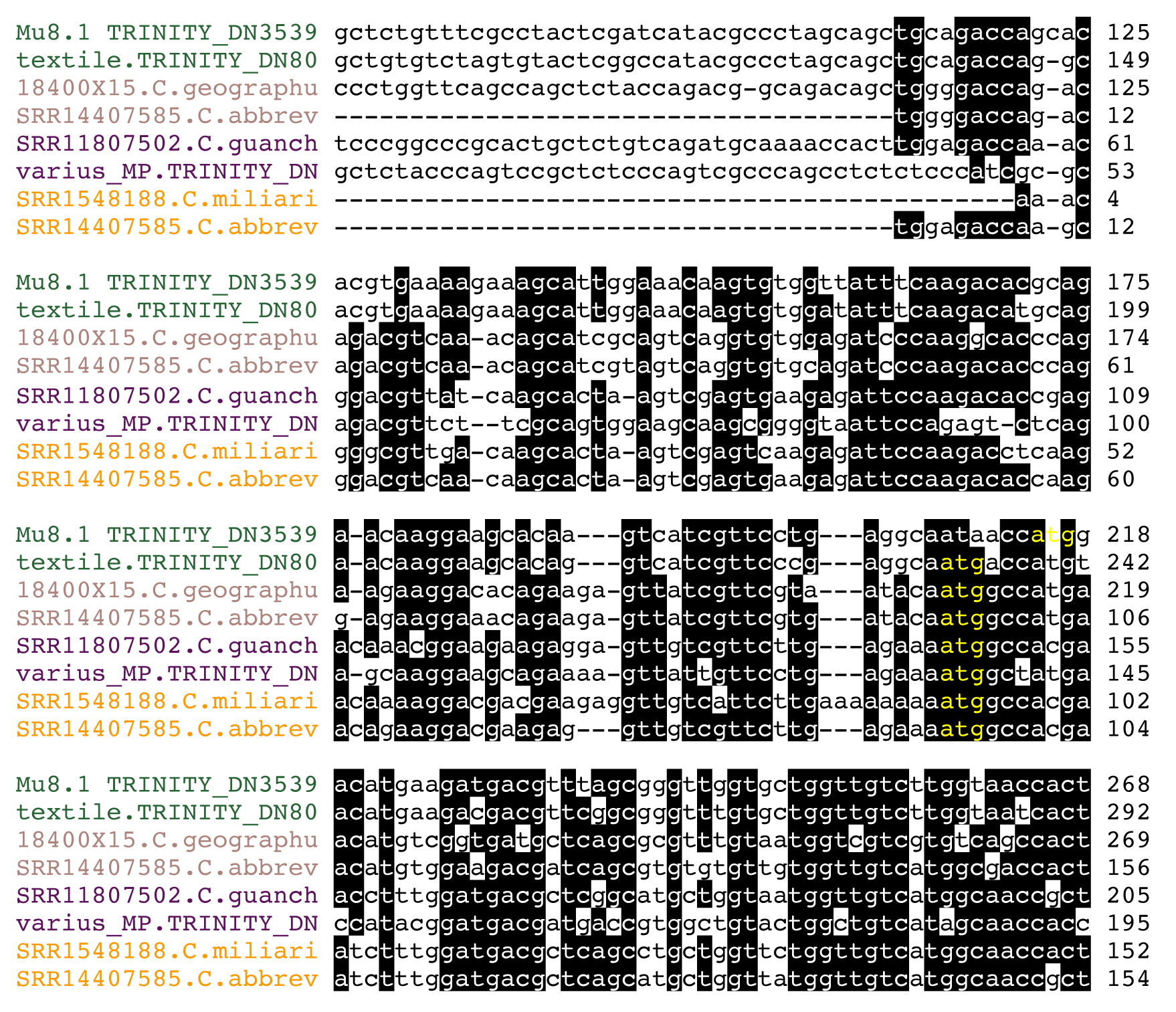

Supplement: S3 Fig — Only the 5′ UTRs and the beginning of ORFs are shown. The start codon is shown in yellow, and columns with ≥75% sequence identity are highlighted in black. The sequence names are colored to match the clusters in S2 Fig. (TIF) [file pbio.3002217.s003.tif]

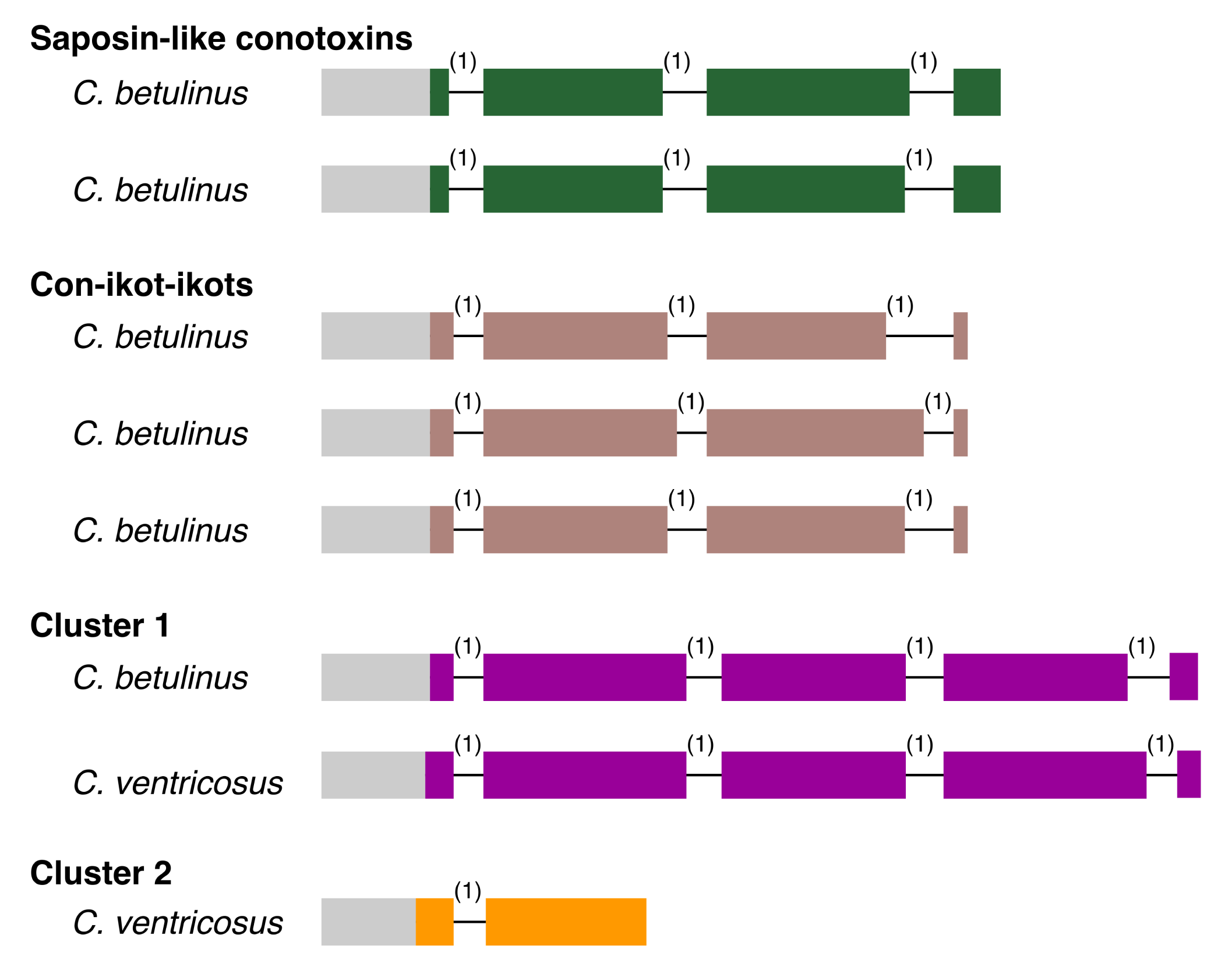

Supplement: S4 Fig — Two transcripts from C. ventricosus and 6 from C. betulinus were identified that could be successfully mapped to the respective genomes. These transcripts were used to assess intron locations and phases from each of the 4 clusters. The exons are represented by wide boxes proportional to the length of the sequences, whereas the introns are shown by thin interspaced segments (not proportional to sequence length) with their phases given above each intron. The predicted signal sequence is colored grey, and the remaining precursor colored to match S2 Fig. (TIF) [file pbio.3002217.s004.tif]

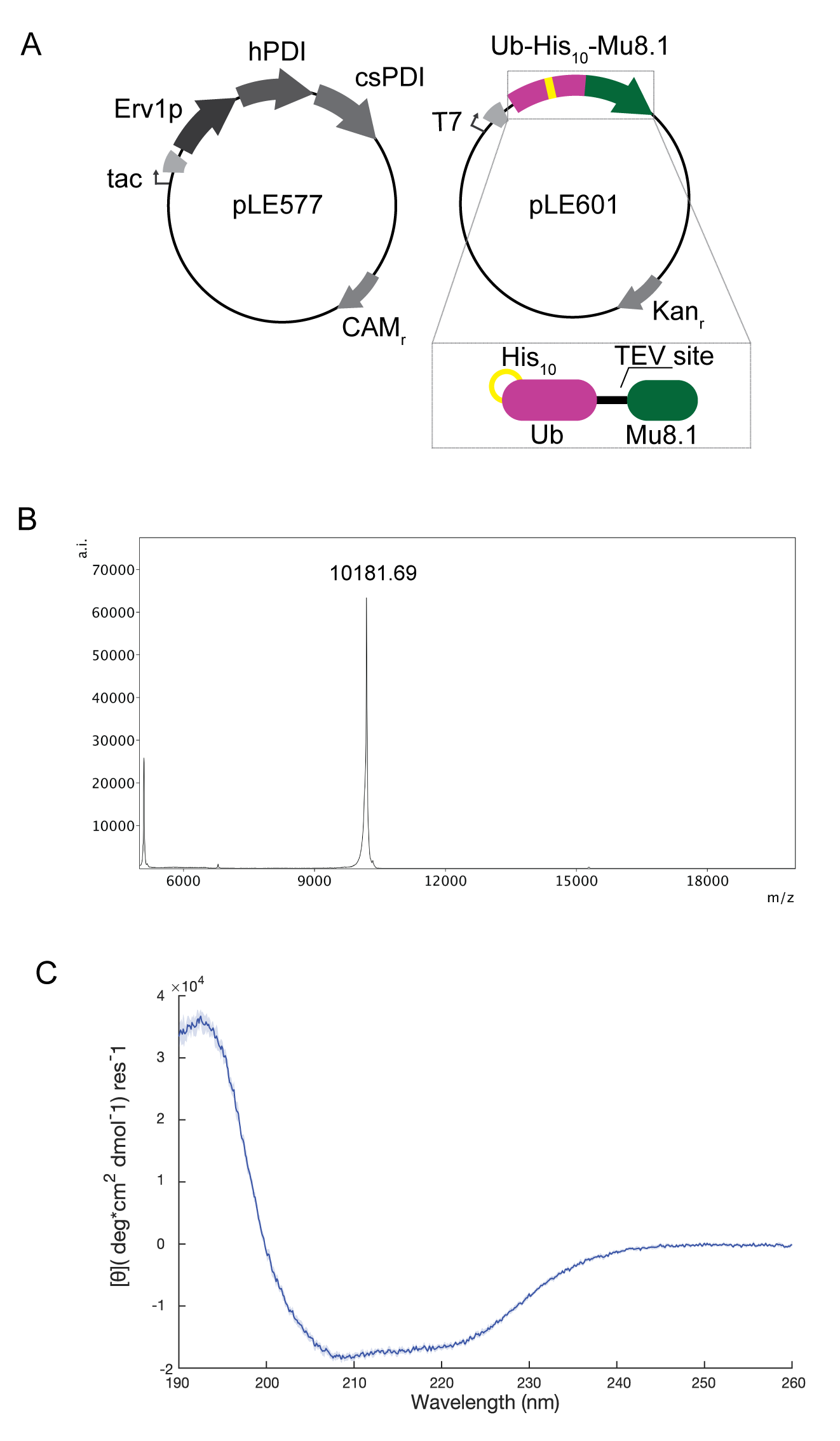

Supplement: S5 Fig — (A) Recombinantly expressed Mu8.1 purifies as a single, fully oxidized species from E. coli. Schematic overview of the 2 plasmids of the csCyDisCo expression system [25] used for recombinant expression of Ub-His10-Mu8.1. The csCyDisCo plasmid (pLE577) encodes for the 3 enzymes, Erv1p, hPDI, and csPDI. pLE601 encodes the Ub-His10-Mu8.1 fusion protein as shown in the light gray box. (B) MALDI-TOF spectrum of nonreduced Mu8.1 showing a single peak with a mass of 10,181.7 Da. The theoretical average mass of Mu8.1 is 10,181.5 Da. (C) CD spectrum of nonreduced Mu8.1 recorded at 25°C. The blue line represents an average of 10 scans. Blue shading around the curve signifies the standard error of the mean of the 10 recorded spectra. CD, circular dichroism; csPDI, conotoxin-specific PDI; hPDI, human PDI; MALDI-TOF, matrix-assisted laser desorption–ionization time of flight; Ub-His10, Ub containing 10 consecutive histidines. (TIF) [file pbio.3002217.s005.tif]

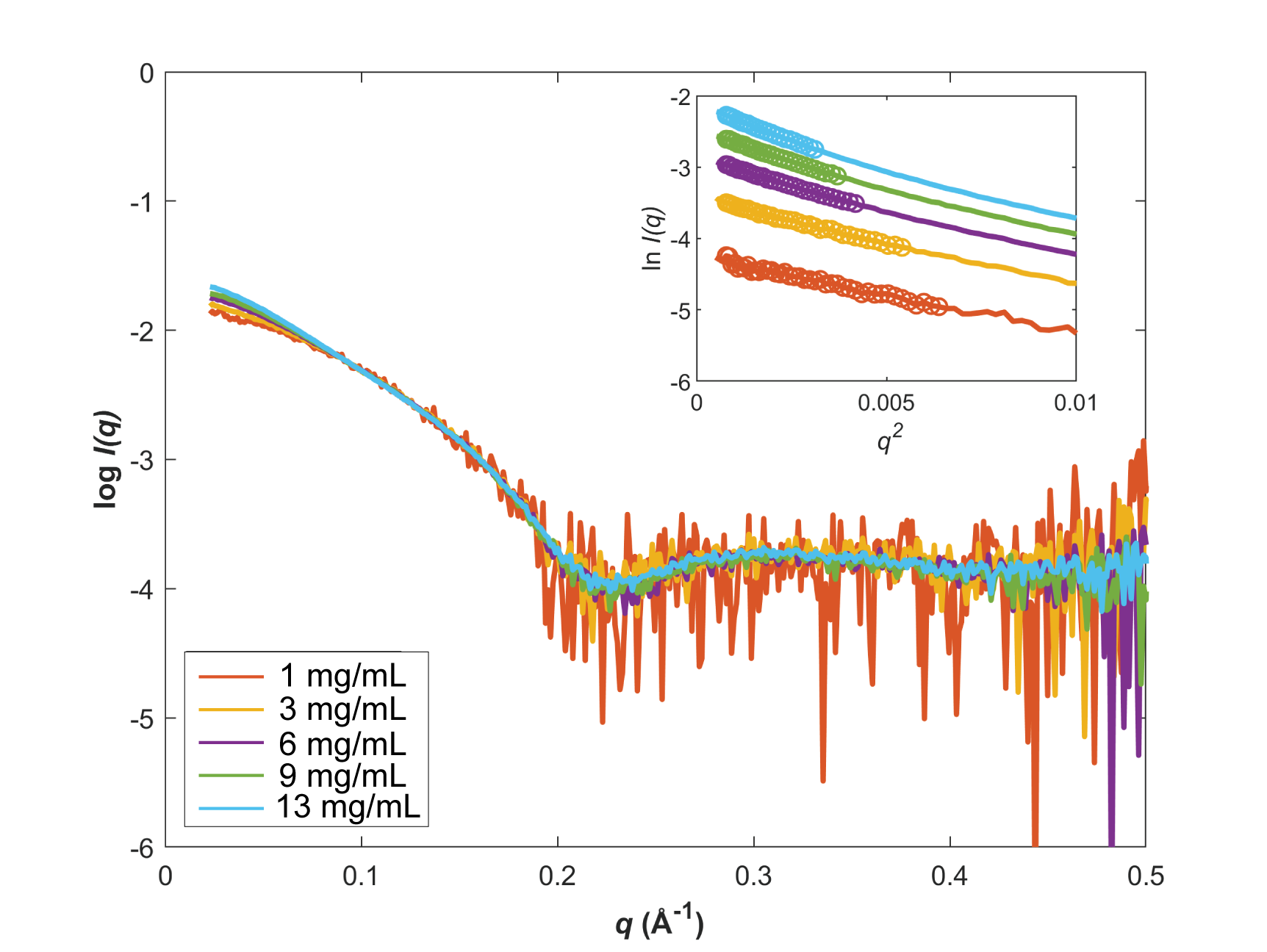

Supplement: S6 Fig — (A) SAXS scattering profiles of increasing concentrations of Mu8.1 dissolved in 10 mM NaPi (pH 8), 150 NaCl. Inset: Guinier plots of scattering profiles, where straight lines were obtained by linear regression of the scattering profiles in the low q2 region. The Guinier region is highlighted with circles. Source data for quantifications provided in S2 Data. (TIF) [file pbio.3002217.s006.tif]

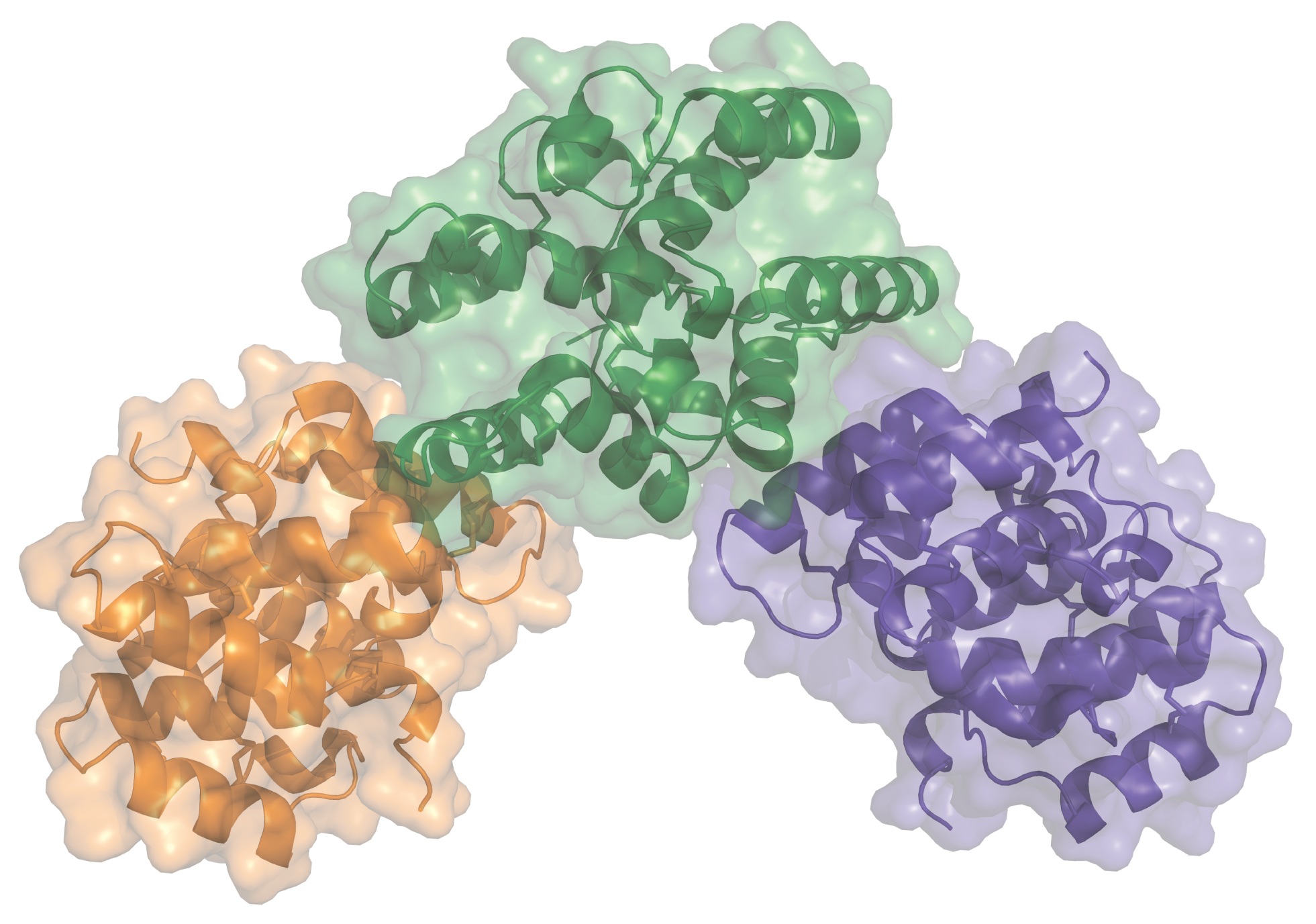

Supplement: S7 Fig — The asymmetric unit of Mu8.1_59 accommodates 6 molecules that form 3 equivalent dimers. The dimers are shown in orange, green, and dark purple. (TIF) [file pbio.3002217.s007.tif]

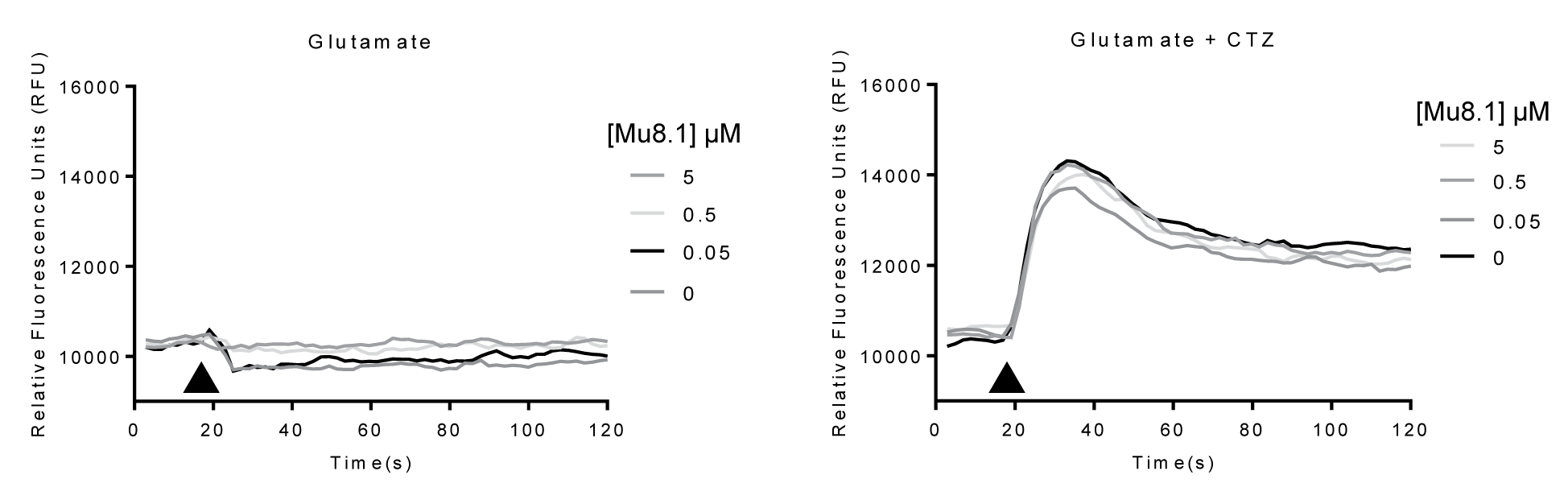

Supplement: S8 Fig — Intracellular Ca2+ imaging was used to determine a possible effect of Mu8.1 on the AMPA receptor GluA2 as described in Materials and methods. The experiment was performed in the absence (left) and presence (right) of cyclothiazide (CTZ), a positive allosteric modulator of AMPA receptors, known to block receptor desensitization and thus increase AMPA receptor current. Black arrows indicate the addition of saturating agonist solution (1 mM glutamate). The experiment was conducted at different concentrations of Mu8.1 as indicated. No effect of Mu8.1 treatment was observed. Source data provided in S5 Data. (TIF) [file pbio.3002217.s008.tif]

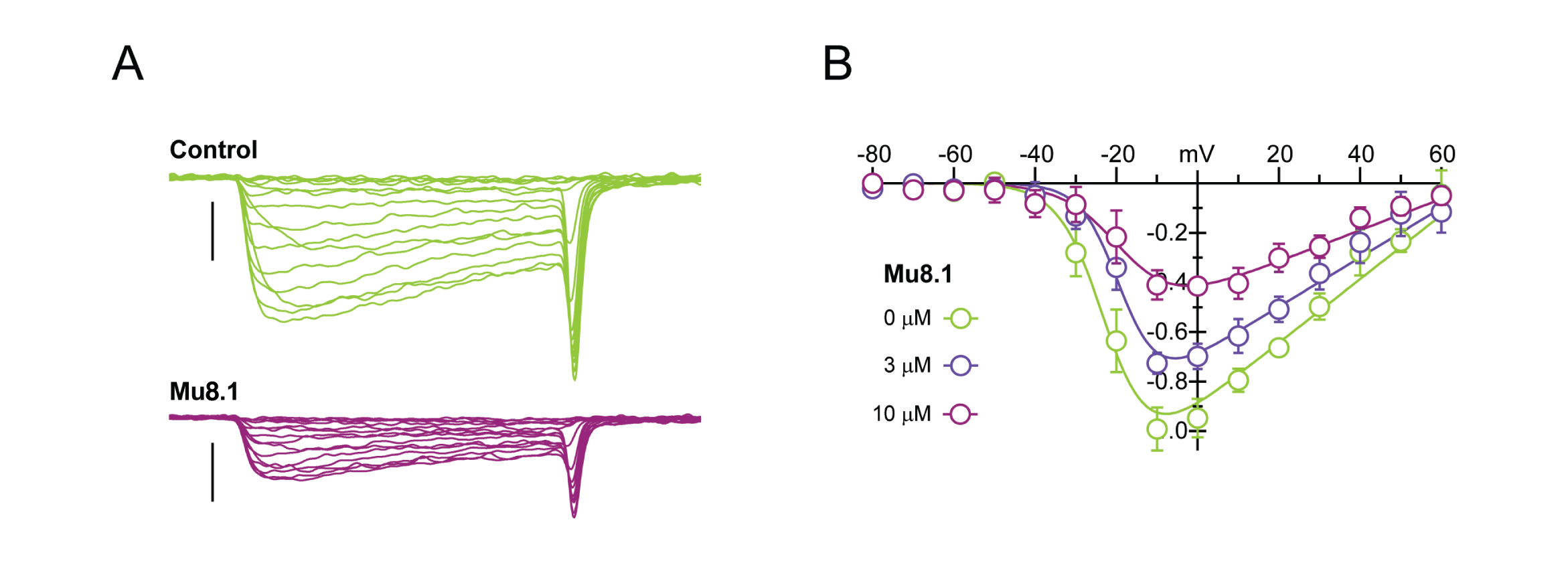

Supplement: S9 Fig — (A) Representative family of Cav2.3 currents elicited by a standard IV protocol (50 ms pulses from −80 mV to 60 mV in 10 mV steps, Vh −90 mV) in the absence (control, green) and the presence of Mu8.1 (10 μM, mauve). Scale bar is 2 nA. (B) Average I-V plots from peak currents in control (′ V0.5 = −22.1 ± 2.4 mV, n = 4) and Mu8.1 (′ 3 μM V0.5 = −18.5 ± 1.9, n = 4; and ′ 10 μM V0.5 = −18.0 ± 2.5, n = 4). One-way ANOVA with Dunnett multiple comparisons test of control vs. 3 μM Mu3.1 p = 0.4402; control vs. 10 μM p = 0.3587. Source data and quantifications provided in S6 Data. (TIF) [file pbio.3002217.s009.tif]

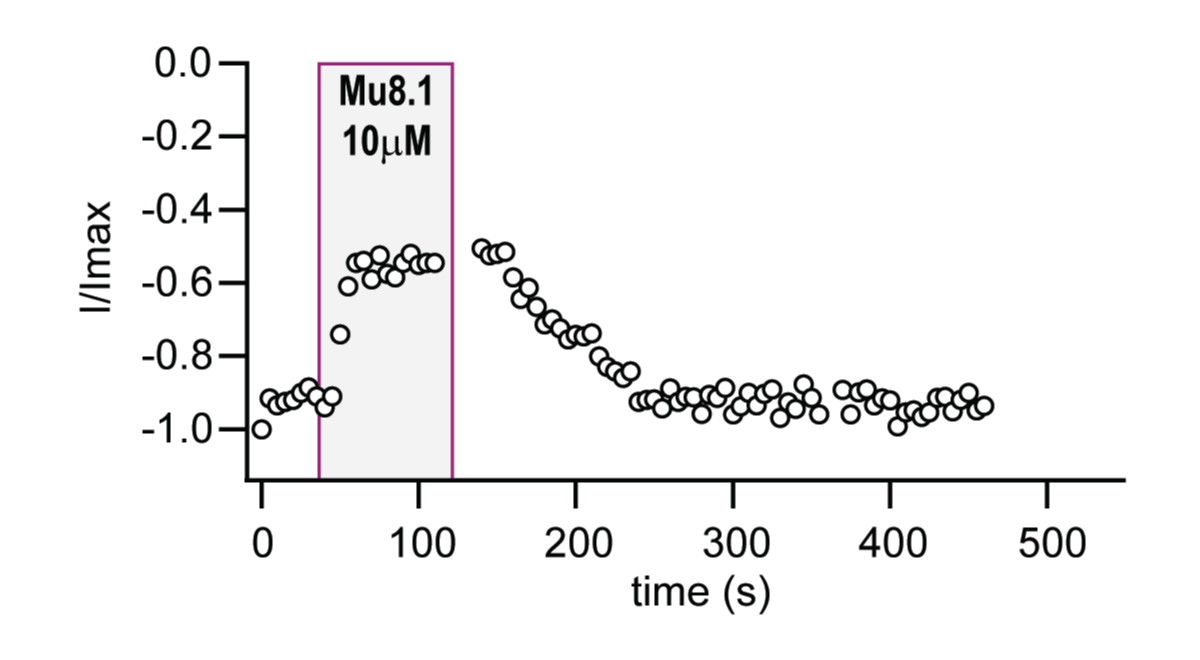

Supplement: S10 Fig — (A) Representative APC whole-cell peak currents recorded from a cell expressing Cav2.3 channels during application of Mu8.1 (10 μM, shaded box) and upon full extracellular bath solution washout. Stimuli: 50 ms pulses to −20 mV at 0.2 Hz (Vh −90 mV). Source data provided in S7 Data. (TIF) [file pbio.3002217.s010.tif]

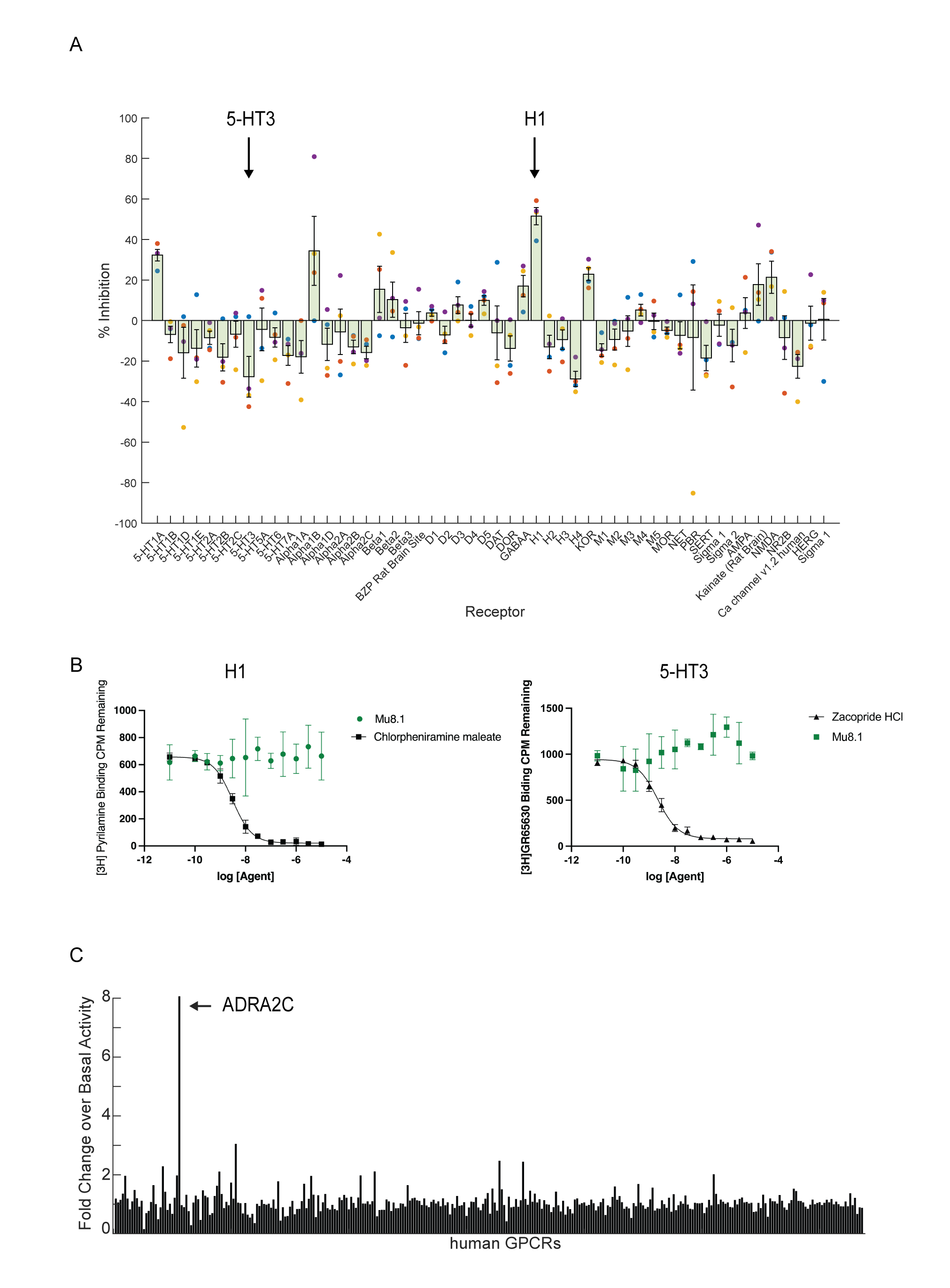

Supplement: S11 Fig — (A) Primary radioligand binding assay of Mu8.1 (10 uM) at 52 receptors and ion channels (x-axis). Plotted values represent means (n = 4), and error bars are ±SEM. Arrows show 2 receptors, 5-HT3 and histamine H1, with inhibition >50% that were selected for secondary testing. Source data and quantifications provided in S8 Data. (B) Secondary binding assay of the 5-HT3 (left) and H1 (right) receptors. Plotted values represent means (n = 3), and error bars are ±SEM. The results demonstrate that the 2 initial hits from S11A were false positives. (C) PRESTO-Tango screen of Mu8.1 (10 μM) at 318 human GPCRs (x-axis). Plotted values represent means ±SD. The arrow indicates a potential hit (ADRAC2) that met the cutoff for further testing. However, a closer examination of the data showed a false positive due to an outlier. (TIF) [file pbio.3002217.s011.tif]

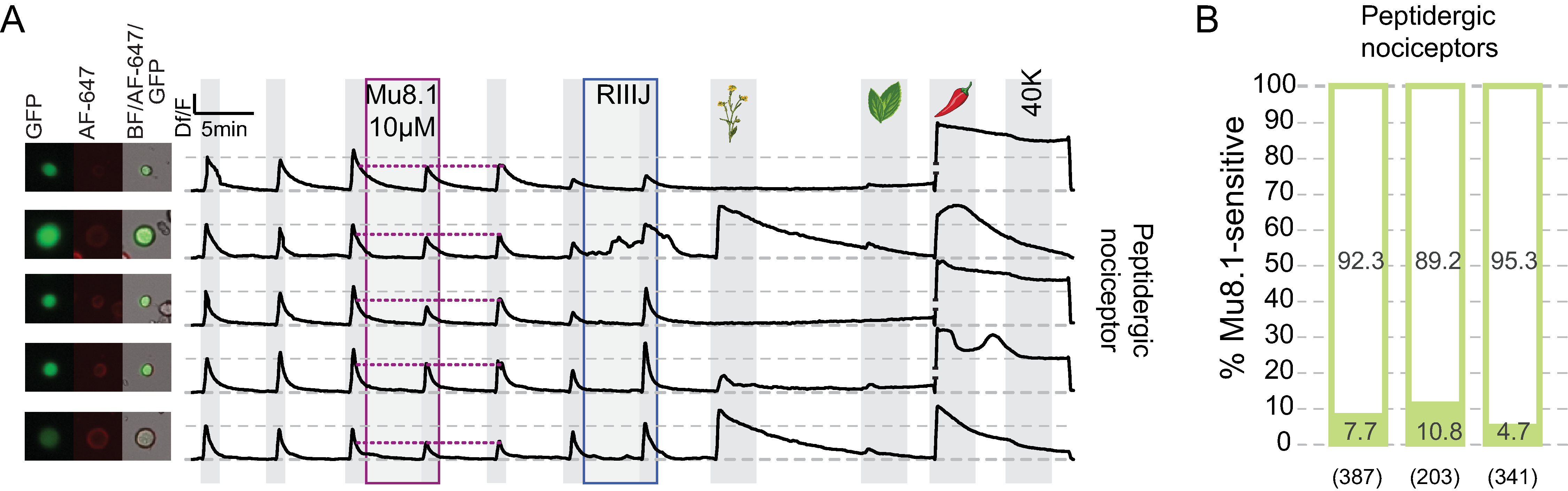

Supplement: S12 Fig — (A) Examples of calcium traces from peptidergic nociceptors in which Mu8.1 inhibition of the peak was not reversed upon washout. Treatment with 10 μM Mu8.1 was scored as “irreversible” if the second Ca2+ peak after Mu8.1 treatment was lower than the peak immediately before Mu8.1 treatment (see mauve dotted line). Each trace represents the calcium signal (ΔF/F) of the neuron pictured on the left (GFP-CGRP+: peptidergic nociceptors; Alexa Fluor 647-Isolectin B4+: nonpeptidergic nociceptors, and bright-field). KCl depolarization pulses (25 mM) are indicated by light grey shading. A higher KCl pulse (40 mM) was used to elicit a maximum calcium signal at the end of the experiment. Class-defining pharmacology: RIIIJ (1 μM, blue box); AITC (100 μM; mustard flower), menthol (400 μM; peppermint leaf), and capsaicin (300 nM; chili pepper). Horizontal lines flanking breaks within a trace signify graphical adjustment of trace amplitude to avoid overlap of neighboring traces. (B) Peptidergic nociceptor populations from 3 independent experiments showing the percentage of neurons (within the bars) where Mu8.1 treatment was reversible (empty) or deemed irreversible (filled). The number of peptidergic nociceptive neurons recorded in each experiment is shown in parentheses. Source data of individual traces and for quantifications shown in S9 Data. AITC, allyl isothiocyanate; CGRP, calcitonin gene-related peptide; GFP, green fluorescent protein. (TIF) [file pbio.3002217.s012.tif]

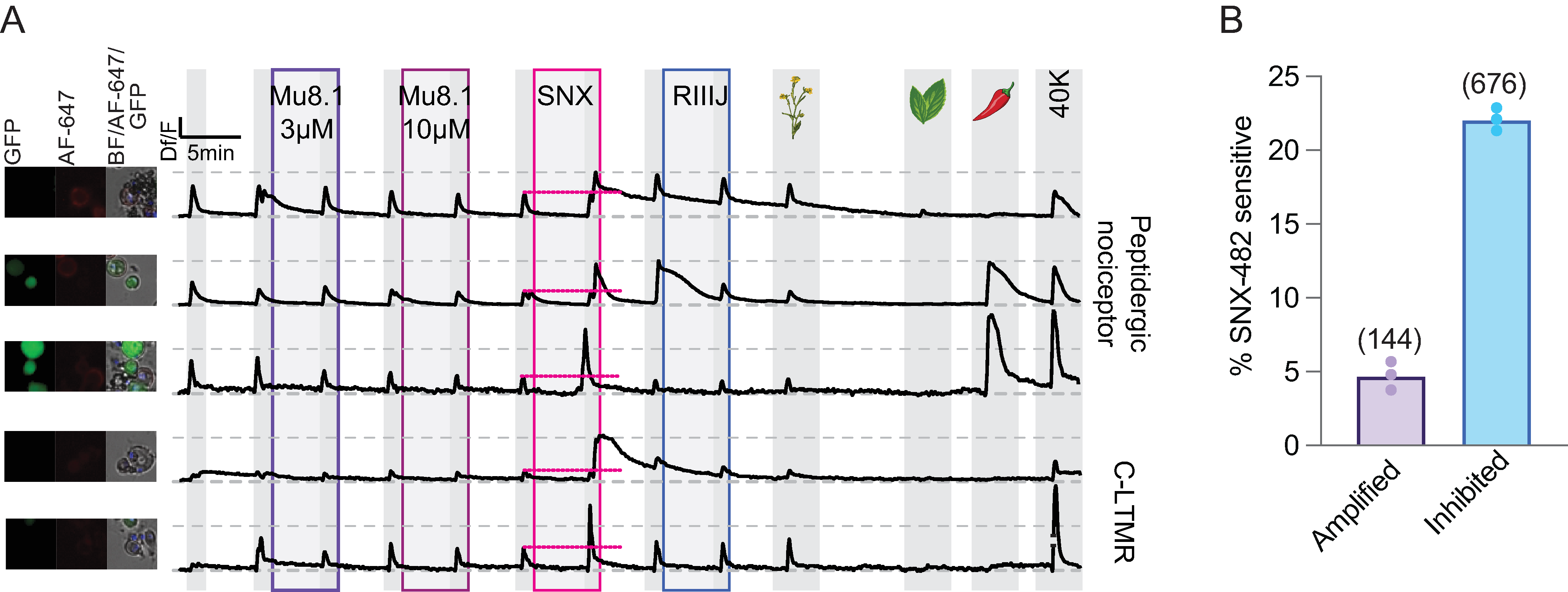

Supplement: S13 Fig — Examples of calcium traces from peptidergic nociceptors and C-LTMRs in which SNX-482 amplified the KCl depolarization-induced Ca2+ peak (see pink dotted line). Each trace represents the calcium signal (ΔF/F) of the neuron pictured on the left (GFP-CGRP+: peptidergic nociceptors; Alexa Fluor 647-Isolectin B4+: nonpeptidergic nociceptors, and bright-field). KCl depolarization pulses (25 mM) are indicated by light grey shading. A higher KCl pulse (40 mM) was used to elicit a maximum calcium signal at the end of the experiment. Class-defining pharmacology: RIIIJ (1 μM, blue box); AITC (100 μM; mustard flower), menthol (400 μM; peppermint leaf), and capsaicin (300 nM; chili pepper). Horizontal lines flanking breaks within a trace signify graphical adjustment of trace amplitude to avoid overlap of neighboring traces. (B) Neurons affected by SNX-482 from 3 independent experiments showing the percentage of neurons that were amplified or inhibited by SNX-482. The number of neurons in each experiment is shown in parentheses. Source data of individual traces and quantification provided in S10 Data. AITC, allyl isothiocyanate; C-LTMR, C-low threshold mechanoreceptor; CGRP, calcitonin gene-related peptide; GFP, green fluorescent protein. (TIF) [file pbio.3002217.s013.tif]

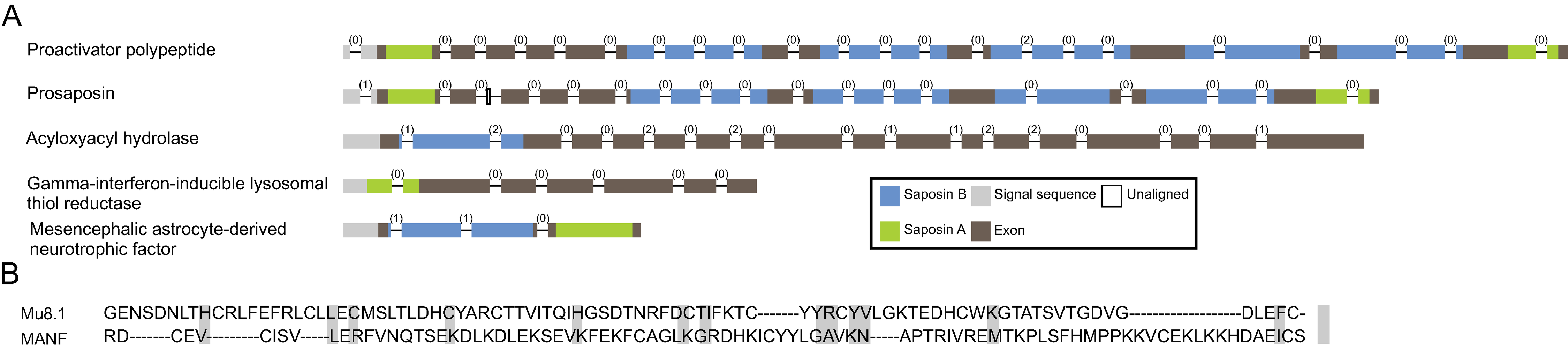

Supplement: S14 Fig — (A) Gene structure of saposin domain-containing proteins in C. ventricosus. The exons are represented by wide boxes proportional to the length of the sequences, whereas the introns are shown by thin interspaced segments (not proportional to sequence length) with their phases given above each intron. (B) Sequence alignment of the saposin domains from Mu8.1 and C. ventricosus mesencephalic astrocyte-derived neurotrophic factor (MANF) revealing only little sequence similarity. Source data provided Supplementary_file_D.saposins in S1 Data. (TIF) [file pbio.3002217.s014.tif]

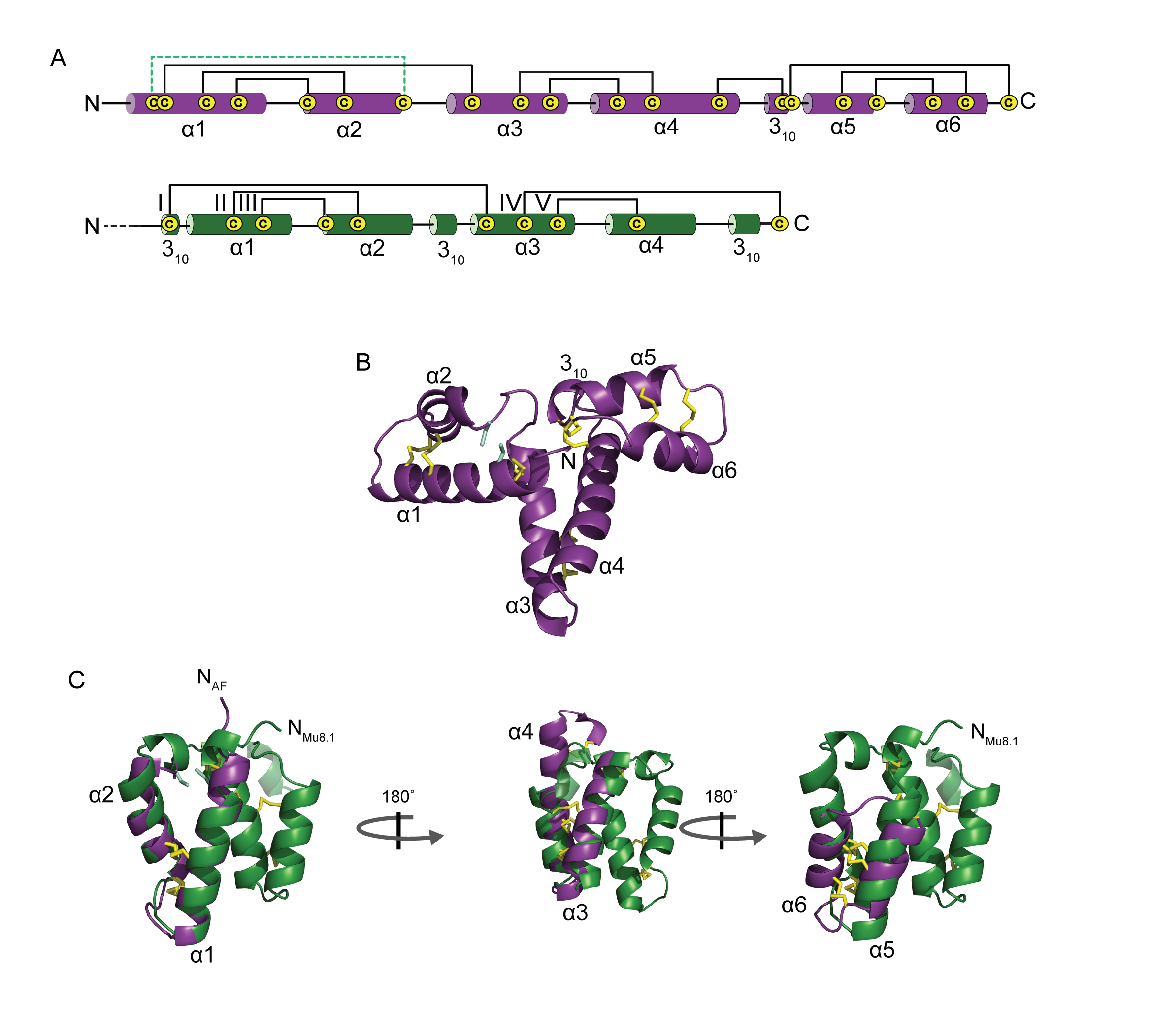

Supplement: S15 Fig — (A) Graphic representation of AlphaFold-predicted secondary structure elements and disulfide bonds of a Cluster 1 conotoxin from C. litteratus (sequence found in Supplementary file A in S1 Data and [81]) (purple) compared to Mu8.1 (green). Disulfide bonds are represented by brackets, and α- and 310-helices are represented as cylinders (numbered by Arabic numerals). The dotted pale green bracket represents a putative disulfide bond not predicted by AlphaFold. (B) Cartoon representation of the structure predicted byAlphaFold for the Cluster 1 toxin. The predicted structure displays an α-helical protein with 3 leaf-like domains each consisting of a helix–turn–helix motif. Disulfide bonds are represented by yellow sticks, and free cysteines are represented by pale green sticks. (C) The 3 helix–turn–helix motifs individually overlaid with the Mu8.1 crystal structure. Note the close structural similarity (and disulfide pattern) between the first 4 helices of the Cluster 1 protein and Mu8.1, apart from a predicted different orientation between the 2 helix–turn–helix motifs. (TIF) [file pbio.3002217.s015.tif]

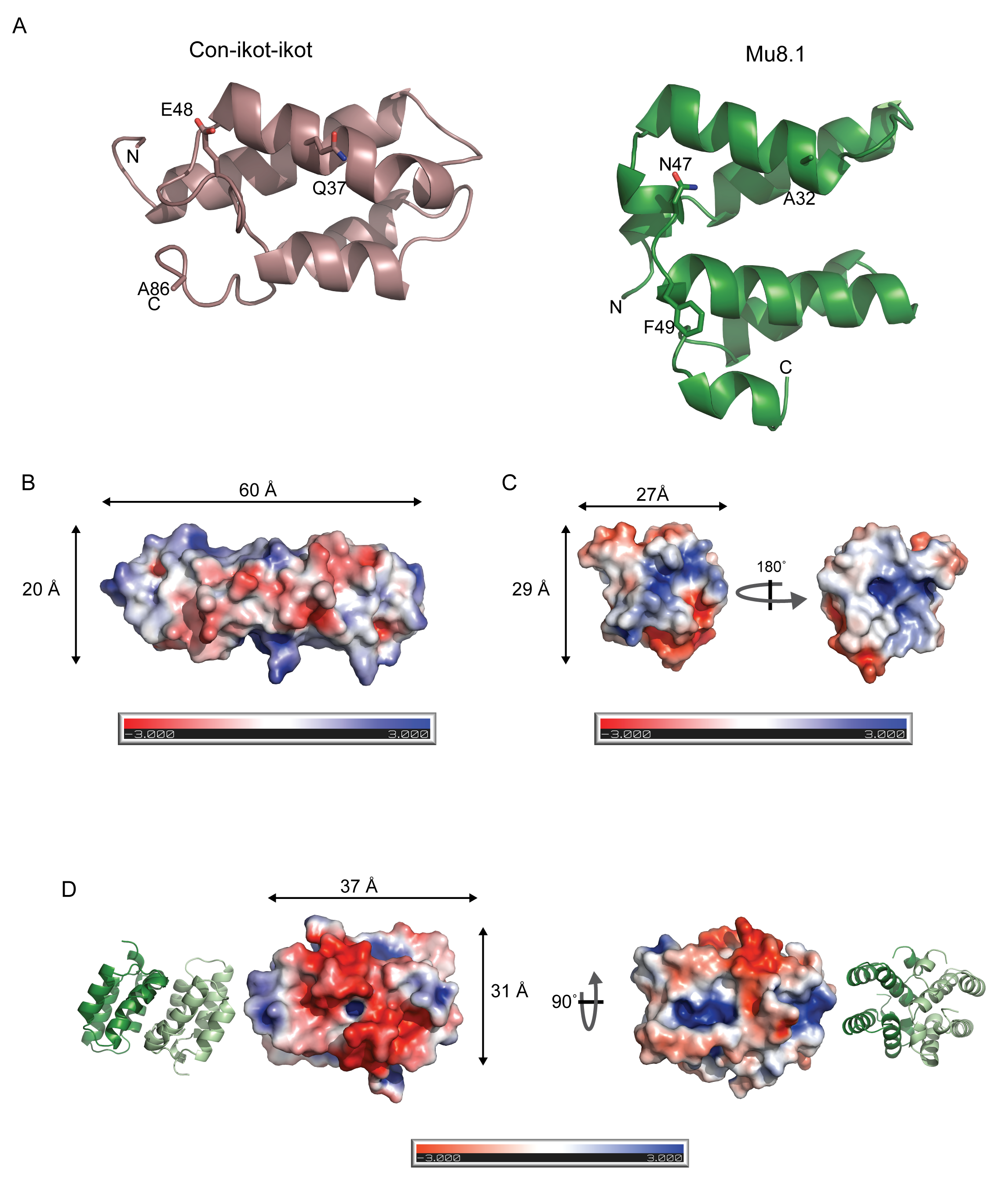

Supplement: S16 Fig — (A) The GluA2 AMPA receptor-binding surface of con-ikot-ikot with residues important for binding [29] shown as stick models (left) and the corresponding surface and residues in the Mu8.1 protomer (right). (B) Electrostatic surface representation (red: negative; blue: positive) of the con-ikot-ikot GluA2 AMPA receptor-binding surface [29]. (C) Electrostatic surface representation of the Mu8.1 protomer—left: the outer surface of the dimer; right: the surface of the dimer interface. (D) Electrostatic surface representation of the Mu8.1 dimer showing a negatively charged patch (left). The cartoon representations next to each surface representation indicate orientation. (TIF) [file pbio.3002217.s016.tif]

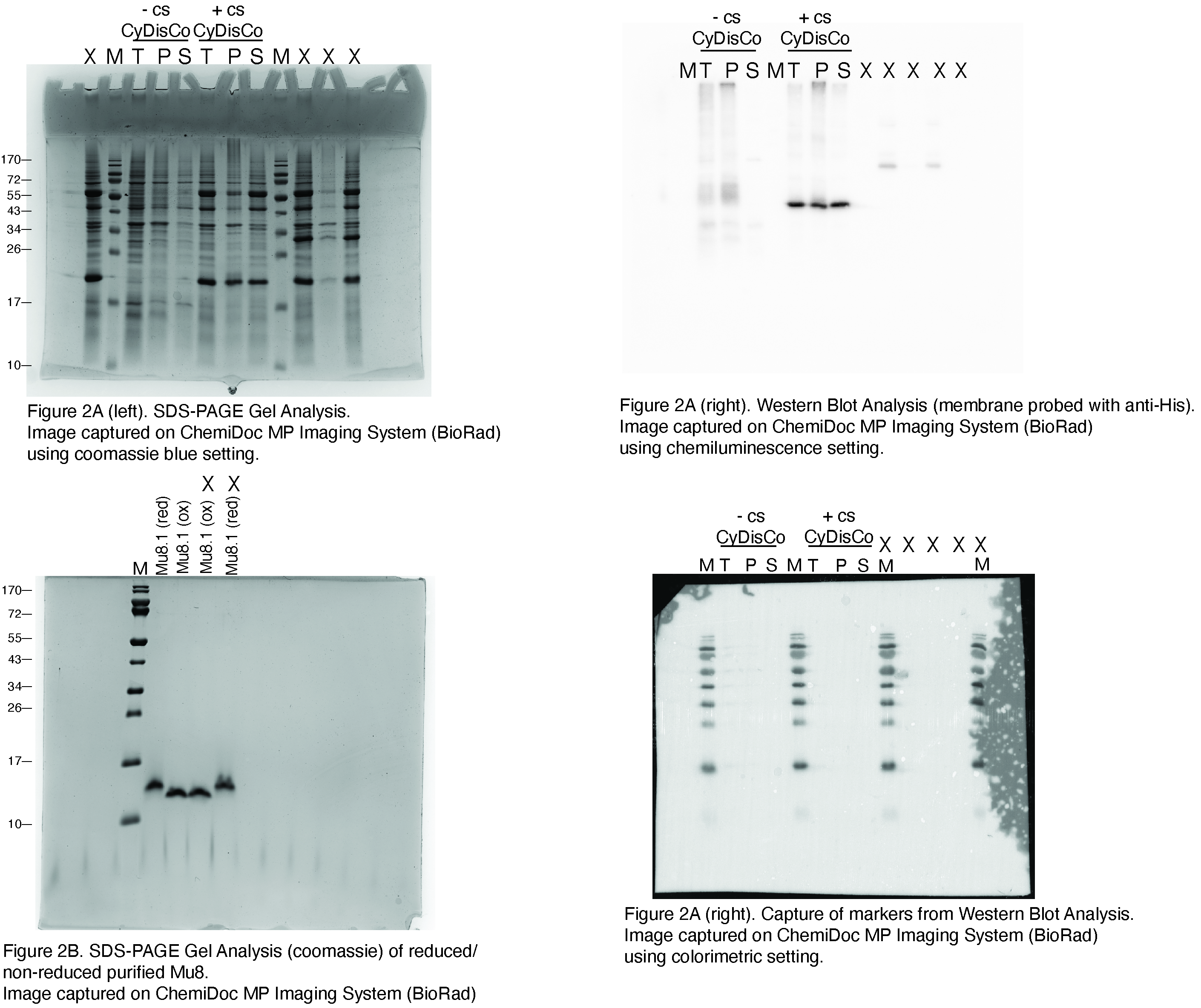

Supplement: S1 Raw Images — (TIF) [file pbio.3002217.s031.tif]
