# Supplementary material for: A previously unrecognized superfamily of macro-conotoxins includes an inhibitor of the sensory neuron calcium channel Cav2.3
Source: PLoS Biol. 2023 Aug 3;21(8):e3002217. doi: 10.1371/journal.pbio.3002217 (PMC10437998; doi:10.1371/journal.pbio.3002217)
Supplement: S1 Table — (PDF) [file pbio.3002217.s017.pdf]

S1 Table. Available venom gland transcriptome datasets searched for Mu8.1- and con-ikot-ikot-like sequences

Conus species	Accession numbers
Conus epsicopatus	DRX030966, SRR6983169, SAMD00029746
Conus raulsilvai	SRR11807492
Conus infinitus	SRR11807493
Conus antoniomonteiroi	SRR11807494
Conus miruchae	SRR11807495
Conus cuneolus	SRR11807496
Conus boavistensis	SRR11807497
Conus verdensis	SRR11807498
Conus galeao	SRR11807500
Conus maioensis	SRR11807501, SRR11807499
Conus guanche	SRR11807502
Conus grahami	SRR11807507
Conus ventricosus	SRR13740844
Conus bayani	SRR13781584
Conus ebraeus	SRR14407576, SRR14407590, SRR2609538
Conus mordeiraeo	SRR14407578, SRR14407579
Conus regonae	SRR14407580, SRR14407581
Conus fulgetrum	SRR14407582
Conus abbreviatus	SRR14407584, SRR14407585
Conus aristophanes	SRR14407586, SRR14407587
Conus judaeus	SRR14407589
Conus coronatus	SRR14407591, SRR14407592, SRR2609545
Conus miliaris	SRR1542424, SRR1542681, SRR1544117, SRR1544118, SRR1544119, SRR1544120, SRR1544137, SRR1544140, SRR1544142, SRR1544595, SRR1544597, SRR1544600, SRR1544622, SRR1544627, SRR1544690, SRR1544692, SRR1548185, SRR1548186, SRR1548187, SRR1548188, SRR1548189, SRR1548190
Conus betulinus	SRR2124881
Conus ermineus	SRR6983161, SRR6983162, SRR6983163, SRR6983164, SRR6983165, SRR6983166, SRR6983167, SRR6983168, SRR6983169
Conus magus	SRR8195628, SRR9831255
Conus arenatus	SRR2609544
Conus consors	SRR1954994
Conus gloriamaris	SRR5499408
Conus imperialis	SRR12186674, SRR12186675, SRR12186676, SRR12186677, SRR12186678, SRR12186679, SRR2609542
Conus lividus	SRR2609539
Conus marmoreus	SRR8195632, SRR2609532
Conus quercinus	SRR2609537, CNS0048932
Conus rattus	SRR2609540
Conus rolani	SRR16493597

Conus sponsalis	SRR2609541
Conus striatus	SRR8195630
Conus terebra	SRR8195627
Conus textile	SRR8195629
Conus tribblei	SRR1799982
Conus varius	SRR2609543
Conus virgo	SRR8195631, SRR2608262
Conus geographus	SRR503416, SRR503415, SRR503414, SRR503413
Conus trochulus	SRR11807506
Conus reticulatus	SRR11807504
Conus caracteristicus	CNS0048931
Conus generalis	CNS0048933
