# Supplementary material for: A previously unrecognized superfamily of macro-conotoxins includes an inhibitor of the sensory neuron calcium channel Cav2.3
Source: PLoS Biol. 2023 Aug 3;21(8):e3002217. doi: 10.1371/journal.pbio.3002217 (PMC10437998; doi:10.1371/journal.pbio.3002217)
Supplement: S2 Table — Data extraction was performed using RAW. Rg Guinier; radius of gyration (Rg) calculated from the slope of the Guinier plot (Panel A, inset); Rg P(r), radius of gyration obtained from pairwise distribution function; I(0)guinier, intensity at zero scattering angle extrapolated from Guinier plot; I(0) P(r), intensity at zero scattering obtained from pairwise distribution function; Dmax, maximum dimension; Mw Bayes, molecular weight from Bayesian inference; Mw Vc, molecular weight from volume of correlation; Primus scale to 1 mg/mL sample, scaling obtained using the Primus program from the ATSAS package [82]; Est concentration from scale, estimated concentration from Primus scale in mg/ml; Mw from scaled concentration (conc), molecular weight as a function of concentration. Source data for quantifications provided in S2 Data. (B) Percentages of oligomeric species in solution at different concentrations of Mu8.1. The percentages of oligomeric species from monomer to octamer were determined with Oligomer [69] from the ATSAS package [82]. Source data for quantifications provided in S2 Data. (PDF) [file pbio.3002217.s018.pdf]

S2 Table

Table A

Sample	Rg, Guinier	Rg P(r)	I(0) Guinier	I(0) P(r)	Dmax	Mw Bayes	Mw Vc	Primus scale to 1 mg/mL sample	Est concentration from scale	Mw from scaled conc
1 mg/mL	18.6	17.1	0.0145	0.0137	45	18.1	18.9	1	1.00	20
3 mg/mL	20.4	20.6	0.051	0.0507	65	18.7	20.6	0.3231	3.10	22.8
6 mg/mL	22.7	23.3	0.1214	0.1216	80	23.7	23.7	0.1584	6.31	26.7
9 mg/mL	23.8	24.1	0.1786	0.1779	79	25.6	25.8	0.1175	8.51	29.1
13 mg/mL	25.5	26.2	0.3143	0.315	89	28.9	28.7	0.0755	13.25	32.7

Table B

Sample	Concentration mg/mL	Monomer %	Dimer %	Tetramer %	Octamer %	χ^2
1 mg/mL	1	0	85	15	1	0.94
3 mg/mL	3.1	0	72	25	4	0.96
6 mg/mL	6.3	0	57	37	6	1.03
9 mg/mL	8.5	0	47	46	8	1.14
13 mg/mL	13.2	0	34	55	12	1.15
