# Supplementary material for: A previously unrecognized superfamily of macro-conotoxins includes an inhibitor of the sensory neuron calcium channel Cav2.3
Source: PLoS Biol. 2023 Aug 3;21(8):e3002217. doi: 10.1371/journal.pbio.3002217 (PMC10437998; doi:10.1371/journal.pbio.3002217)
Supplement: S3 Table — Automated patch clamp single concentration Mu8.1 screening of recombinantly expressed ion channels. Data for Cav2.3 are provided in Fig 7. Mu8.1 concentrations, test pulse, and IC50* estimates from single concentration experiments for each channel tested. IC50* = {fc/(1-fc)} × [Mu8.1]; fc = IMu8.1/ICtr; fc: fractional current; IMu8.1: current in the presence of Mu8.1; ICtr: current in the absence of toxin. NB = no block; SEM = standard error of the mean; n = number of experiments. Source data are provided in S4 Data. (PDF) [file pbio.3002217.s019.pdf]

S3 Table

Ion Channel	Test Potential	[Mu8.1]	Inhibition			IC_{50}*
	(mV)	(μM)	(%)			(μM)
			Mean	SEM	n	
Cav1.2	10	10	NB		5	-
Cav2.1	0	10	22	7	5	35.9
Cav2.2	10	10	12	5	5	73.3
Cav3.1	-20	10	13	5	5	66.9
Cav3.2	-20	10	16	5	5	53.3
Cav3.3	-20	10	24	1	5	32.4
Kv1.1	20	10	19	5	5	42.6
Kv1.2	20	10	6	3	5	168.6
Kv1.3	20	10	NB		6	-
Kv4.3	20	10	NB		5	-
hERG	20#	30	NB		5	-
Nav1.2	-10	30	NB		5	-
Nav1.4	-10	30	NB		5	-
Nav1.7	-10	30	8	2	5	327.1
