# Supplementary material for: A previously unrecognized superfamily of macro-conotoxins includes an inhibitor of the sensory neuron calcium channel Cav2.3
Source: PLoS Biol. 2023 Aug 3;21(8):e3002217. doi: 10.1371/journal.pbio.3002217 (PMC10437998; doi:10.1371/journal.pbio.3002217)
Supplement: S1 File — (DOCX) [file pbio.3002217.s020.docx]

Supplementary Materials and Methods

**A previously unrecognized superfamily of macro-conotoxins includes an inhibitor of the sensory neuron calcium channel Cav2.3**

Celeste M. Hackney^1^, Paula Flórez Salcedo^2^, Emilie Mueller^3^, Thomas Lund Koch^8,9^, Lau D. Kjelgaard^1^, Maren Watkins^4^, Linda Grønborg Zachariassen^5^, Pernille Sønderby Tuelund^5^, Jeffrey R. McArthur^6^, David J. Adams^6^, Anders S. Kristensen^5^, Baldomero Olivera^4^, Rocio K. Finol-Urdaneta^6,7^, Helena Safavi-Hemami^4,8,9^, Jens Preben Morth^3^, Lars Ellgaard^1,^*

Affiliations:

^1^ Department of Biology, Linderstrøm-Lang Centre for Protein Science, University of Copenhagen, Copenhagen, Denmark

^2^ Department of Neurobiology and Anatomy, University of Utah, Salt Lake City, Utah, USA.

^3^ Enzyme and Protein Chemistry, Section for Protein Chemistry and Enzyme Technology, Department of Biotechnology and Biomedicine, Technical University of Denmark, Kgs. Lyngby, Denmark

^4^ School of Biological Sciences, University of Utah, Salt Lake City, Utah, USA.

^5^ Department of Drug Design & Pharmacology, University of Copenhagen, Copenhagen, Denmark

^6^ Illawarra Health and Medical Research Institute (IHMRI), Faculty of Science, Medicine and Health, University of Wollongong, Wollongong, Australia.

^7^ Electrophysiology Facility for Cell Phenotyping and Drug Discovery, Wollongong, Australia.

^8^ Department of Biochemistry, University of Utah, Salt Lake City, Utah, USA.

^9^ Department of Biomedical Sciences, University of Copenhagen, Copenhagen, Denmark.

*Corresponding author: lellgaard@bio.ku.dk

**Materials and Methods**

**Transcriptome mining.** The transcriptomes of 44 species of cone snails publicly available from NCBI, DDBJ, and CNGB repositories were mined with Mu8.1 and coni-ikot-ikot sequences as queries (**Table S1**). The transcriptomes were assembled as described previously (1). Using tBLASTn with e<1e-3 we identified 496 transcripts with sequence similarity to Mu8.1 and con-ikot-ikots. The identified transcripts can be found in S1 Data, file name Supplementary file A.

**CLANS clustering analysis.** To investigate the relationship of the identified toxin sequences, we performed a CLANS clustering analysis (2) using the web tool https://toolkit.tuebingen.mpg.de/tools/clans. CLANS performs an all-against-all BLASTp and retrieves the negative logarithm of the resulting p-values < 1e-6, which are used as attractive forces between the nodes representing the sequences. The additional uniformly repulsive field between all nodes forces the nodes with the lowest blast p-values to cluster together. The BLAST p-values were calculated using the BLOSUM62 matrix. The clustering was first done in 3D for 20,000 rounds and subsequently in 2D for an additional 10,000 rounds, at which point the nodes converged to an equilibrium. The resulting clans-file is supplied in S1 Data, file name Supplementary file B and can be viewed using the command: java -Xmx4G -jar clans.jar -load Supplementary_file_B.clans.jar

**UTR sequence alignment.** We aligned the complete transcripts of Mu8.1, an additional SLC transcript, and two random transcripts from each of the other three toxin clusters using MAFFT L-Ins-I v7.487 and colored the alignment using https://www.bioinformatics.org/sms2/color_align_cons.html. Fig. S3 shows the 5’ UTR and initial segment of the ORF.

**Gene structure and intron sequence alignment.** We identified two transcripts from *Conus ventricosus* and six from *Conus betulinus* that could be mapped in their entire length to the respective genomes. We extracted the 100 intronic nucleotides flanking the ORF encoding exons (S1 Data, Supplementary file C) and aligned the 5’ and 3’ regions separately using MAFFT L-Ins-I v7.487. Supplementary file C shows the percentage sequence identity of the intron regions. The transcripts were mapped onto the genome using Splign v5 (3) to identify the location and phase of the introns.

**Determination of protein oxidation state by MALDI-TOF mass spectrometry (MS)**. Mu8.1 (500 µL) was first desalted by reversed-phase high-performance liquid chromatography (RP-HPLC) chromatography. Trifluoroacetic acid (TFA) was added to a final concentration of 0.1%. The acidified sample was centrifuged at 16,100 g for 15 min and RP-HPLC was performed on an ÄKTA purifier 900 system equipped with a Kromasil C18 column (4.6 mm x 150 mm, 5 µm) using solvent A (5% ethanol, 0.1% TFA) and solvent B (90% ethanol, 0.1% TFA). Elution was executed as a 0% to 75% gradient over 35 mL at a flow rate of 1 mL/min. Protein-containing fractions were analyzed ﻿by MALDI-TOF MS using an Autoflex Smartbeam III instrument (Bruker) calibrated by external calibration (Peptide calibration standard I; Bruker Daltronics). Samples for analysis were mixed with alpha-cyano-4-hydroxycinnamic acid matrix prepared with 70% acetonitrile, 0.1% TFA, spotted on a stainless-steel target plate and analyzed in positive reflectron mode.

**Circular Dichroism (CD) spectroscopy**. CD spectroscopy was performed on a JASCO J-810 CD Spectropolarimeter. Mu8.1 was dissolved in 10 mM sodium phosphate buffer, pH 8 to a final concentration of 10 µM Mu8.1 in 200 µL buffer. CD spectra were recorded in a 0.1 cm cuvette at 25°C in the wavelength range of 190 nm to 260 nm using a data pitch of 0.1 nm and a bandwidth of 1.0 nm. The final spectra were obtained by averaging 10 spectra recorded at a scan rate of 10 nm/min and the baseline (buffer only) was subtracted. The measured ellipticity was then converted to residual molar ellipticity and the data were visualized using Matlab (MathWorks).

**Intracellular Ca^2+^ imaging for determination of the effect of Mu8.1 on ionotropic glutamate receptor subtype AMPA receptors**. Human embryonic kidney (HEK) 293T cells (American Type Culture Collection, Manassas, VA) were transiently transfected with rGluA2(Q)i plasmid DNA using LipdoD293™ DNA In Vitro Transfection reagent by following the protocol supplied by the manufacturer. Briefly, HEK293T cells in suspension were mixed with DNA/transfection complex (formed by mixing plasmid DNA, LipdoD293 reagent, and DMEM in a 1:2:25 ratio) and plated into poly-D-lysine–coated Falcon black clear-bottom 96-well plates (Corning, Corning, NY) to a final density of approximately 20,000 cells and 0.025 µg plasmid DNA per well. The competitive antagonist CNQX was added at a final concentration of 20 µM to protect against glutamate-induced cytotoxicity. Cells were incubated for 2 days after transfection before experiments. The intracellular calcium concentration, serving as an indirect measure of receptor activation, upon agonist application, was measured as a change in fluorescence of Fluo-8 AM using a FlexStation I plate reader (Molecular Devices). The experiment was performed in the presence and absence of cyclothiazide (CTZ), a positive allosteric modulator of AMPA receptors, known to block receptor desensitization and thus increase AMPA receptor current (4). On the day of experiments, transfected cells were washed three times in Phosphate Buffered Saline with Ca^2+^ and Mg^2+^ (in mM: 137 NaCl, 2.7 KCl, 10 Na_2_HPO_4_, 2 KH_2_PO_4_, 0.1 CaCl_2_, 0.5 MgCl_2_, pH 7.4) and loaded with a solution containing 2 µM Fluo-8 AM fluorescent indicator dye (dissolved in plain DMEM) and incubated for 30 min at 37 °C. Excess loading dye was removed by washing three times in FLUO buffer (in mM: 140 choline chloride, 5 KCl, 1 MgCl_2_, 10 CaCl_2_, and 10 HEPES pH 7.4). Cells were pre-incubated with 50 µl FLUO buffer containing various concentrations of Mu8.1 in the presence and absence of CTZ for 30 min at room temperature. Changes in dye fluorescence upon addition of a saturating agonist solution with various concentrations of Mu8.1 in the presence and absence of CTZ were measured at 538 nm using excitation at 485 nm. Final concentrations used in the experiment were 1 mM glutamate (agonist), 100 µM CTZ and 0.05, 0.5, and 5 µM Mu8.1. The experiment was performed in quadruplicate wells.

**Initial receptor and GPCRome screening**. Preliminary functional screenings were performed through the National Insititute of Mental Health’s Psychoactive Drug Screening Program (PDSP), contract #HHSN-271-2018-00023-C (NIMH PDSP). The NIMH PDSP is directed by Bryan L. Roth at the University of North Carolina at Chapel Hill and Project Officer Jamie Driscoll at NIMH, Bethesda, MD, USA. Detailed protocols for all assays can be found in the NIMH PDSP Assay Protocol Book accessed via the PDSP website <http://pdsp.med.unc.edu/>.

**Radioligand binding assays**. A primary binding assay was performed on membrane preparations derived from cell lines transiently or stably expressing 52 different receptors (5). Evidence for interaction was based on the inhibition of a reference ligand-binding signal. Secondary binding assays were performed only when Mu8.1 had a signal inhibition greater than 50%. For the primary binding assays, Mu8.1 was applied at a single concentration (10 μM) in quadruplicate in 96-well plates. In secondary binding assays, Mu8.1 was tested in triplicate at eleven concentrations (0.1, 0.3, 1, 3, 10, 30, 100, 300 nM, 1, 3, 10 μM). Both primary and secondary binding assays were carried out in a final volume of 125 μL per well. The “hot” ligand was usually applied at a concentration close to the Kd. Total binding and nonspecific binding were determined in the absence and presence of 10 μM of the appropriate reference compound, respectively. Reactions were stopped by vacuum filtration onto 0.3% polyethyleneimine (PEI) soaked 96-well filter mats using a 96-well Filtermate harvester, followed by three washes with cold wash buffers. Scintillation cocktail was then melted onto the microwave-dried filters on a hot plate and radioactivity was counted in a Microbeta counter.

**GPCR binding assays**. Mu8.1 was also tested on a panel of GPCRs using the PRESTO-Tango system (6). This assay employs HTLA cells, a HEK293T cell-derived stable cell line expressing a human β-arrestin2-TEV protease fusion and a tetracycline-controlled transactivator (tTA)-dependent firefly luciferase reporter gene. Briefly, cells were plated in 384-well plates and incubated overnight. The cells were then transfected with the receptor constructs (318 target receptors) and incubated overnight at 37°C. Following overnight transfection, cells were treated with 10 μM Mu8.1 and incubated overnight at 37°C. Mu8.1 and the media were then removed and Bright-Glo (Promega) reagent was added to determine luciferase activity. Results are presented as fold of the average basal activity. Activity between 0.5 to 2.0-fold of basal is considered normal and not warranting further testing. 100 nM quinpirole stimulation of dopamine receptor DRD2 was used as an assay control.

**Analysis of saposin domain-containing proteins.** 13,594 saposin domain-containing proteins were downloaded from UniProt with the search terms “annotation:(type:"positional domain" saposin)” and clustered with cd-hit v4.8.1 using -c 0.8. The reduced dataset was used as queries against the *C. ventricosus* transcriptome and genome using tBLASTn with an e-value below 1e-10. This search identified five *C. ventricosus* proteins with saposin-like domains (S1 Data, Supplementary file D). The gene structures of the *C. ventricosus* saposin domain-containing proteins were determined similarly as for the toxins.

**AlphaFold structure prediction.** Structural prediction of a randomly selected toxin from Cluster 1 (Supplementary file A (>14718X8.C.litteratus.TRINITY_DN2335_c0_g2_i1_Entry:5131.conotoxin.Conikotikot_len:998_tpm:5563.29 45) and (7)) was generated using the AlphaFold2 (8) implementation in the ColabFold notebook running on Google Colaboratory (9). The predicted structure was produced using the default settings and a custom multiple sequence alignment of mature toxin sequences from Cluster 1. The resulting model was visualized in Pymol. The superposition with Mu8.1 was generated in Pymol.

**References**

1. Ahorukomeye P, Disotuar MM, Gajewiak J, Karanth S, Watkins M, Robinson SD, et al. Fish-hunting cone snail venoms are a rich source of minimized ligands of the vertebrate insulin receptor. eLife. 2019 Feb 12;8:e41574.

2. Frickey T, Lupas A. CLANS: a Java application for visualizing protein families based on pairwise similarity. Bioinformatics. 2004 Dec 12;20(18):3702–4.

3. Kapustin Y, Souvorov A, Tatusova T, Lipman D. Splign: algorithms for computing spliced alignments with identification of paralogs. Biol Direct. 2008;3(1):20.

4. Walker CS, Jensen S, Ellison M, Matta JA, Lee WY, Imperial JS, et al. A Novel Conus Snail Polypeptide Causes Excitotoxicity by Blocking Desensitization of AMPA Receptors. Curr Biol. 2009 Jun;19(11):900–8.

5. Besnard J, Ruda GF, Setola V, Abecassis K, Rodriguiz RM, Huang XP, et al. Automated design of ligands to polypharmacological profiles. Nature. 2012 Dec;492(7428):215–20.

6. Kroeze WK, Sassano MF, Huang XP, Lansu K, McCorvy JD, Giguère PM, et al. PRESTO-Tango as an open-source resource for interrogation of the druggable human GPCRome. Nat Struct Mol Biol. 2015 May;22(5):362–9.

7. Zhang H, Fu Y, Wang L, Liang A, Chen S, Xu A. Identifying novel conopepetides from the venom ducts of Conus litteratus through integrating transcriptomics and proteomics. J Proteomics. 2019 Feb;192:346–57.

8. Jumper J, Evans R, Pritzel A, Green T, Figurnov M, Ronneberger O, et al. Highly accurate protein structure prediction with AlphaFold. Nature. 2021;

9. Mirdita M, Schütze K, Moriwaki Y, Heo L, Ovchinnikov S, Steinegger M. ColabFold - Making Protein folding accessible to all. bioRxiv. 2021;
