# Supplementary material for: A previously unrecognized superfamily of macro-conotoxins includes an inhibitor of the sensory neuron calcium channel Cav2.3
Source: PLoS Biol. 2023 Aug 3;21(8):e3002217. doi: 10.1371/journal.pbio.3002217 (PMC10437998; doi:10.1371/journal.pbio.3002217)
Supplement: Attachment — Submitted filename: Response to reviewers.docx [file pbio.3002217.s032.docx]

**Reviewer #1**

*Reviewer comment:*

*This manuscript describes the isolation and structural characterization of a very large type of conotoxin.  Most of the manuscript deals with the structural aspects which are valuable and novel. Where a little bit more work is needed is on the biophysics and physiology that is also described in the manuscript.*

*Selectivity and electrophysiology:*

*The authors did a nice job in looking at selectivity between Cav2.3 and other types of ion channels, including members of the Cav2 and Cav3 family.   Based on this, I am quite convinced that this molecule is a blocker of this channel, albeit with relatively low 5 micromolar affinity.  What is missing in this though is:*

*IV curve for Cav2.3, and testing whether there is activation gating inhibition*

*time course of development and recovery from block*

*testing the effect of the toxin on a representative member of the Cav1 family*

Answer:

We thank the reviewer for the positive assessment of our manuscript.

The requested data are now added to the revised manuscript as follows:

1) The new Fig. S9 includes in Panel A families of Cav2.3-mediated currents elicited by a standard current-voltage protocol in the absence (control, green) and in the presence of 10 μM Mu8.1 (mauve) and in Panel B average I-V plots from peak current amplitudes measured in control and two different concentrations of Mu.8.1. These plots rendered half activation potentials (V_0.5_) for control and Mu8.1 that are statistically indistinguishable (see figure legend) indicative of the absence of Mu8.1-related activation gating modification of Cav2.3 currents.

2) The new Fig. S10 displays a representative time course of Cav2.3 peak current amplitude change during application of 10 μM Mu8.1 recorded under our experimental conditions. The reduction of peak current during Mu8.1 *wash-in* evidences the development of inhibition, while full extracellular bath solution exchange (*wash-out*) leads to recovery of peak current amplitude near control level. The time constants of inhibition onset (τ_on_ = 16 s) and recovery (τ_off_ = 100 s) in this example render an apparent Kd of ~9 μM, which is consistent with the IC_50_ value of ~6 μM obtained from the concentration-response relationship reported in our manuscript and depicted in Fig. 7B.

3) We have now tested Mu8.1 (10 μM) against human Cav1.2 channels expressed in HEK293 cells (revised Table S4). These experiments failed to reveal modulation of Cav1.2-mediated currents by Mu8.1.

*Reviewer comment:*

Physiology:

The physiological studies that are described are not contextualized properly vis a vis what is know with the role of R-type channels in mice.  Cav2.3 null mice are not sleepy but they show reduced seizure activity in epilepsy models, so it is entirely unclear why the sedative effects were observed here.  It is likely that another target is involved in these processes, and this needs to be discussed in detail.  Along these lines, no proper discussion is provided concerning the zebrafish data, nor is it clear that R-type channels are responsible for the observed effects and the authors do not even try and provide a mechanistic link between R-type block and what is seen in these experiments. In fact, even the title of the figure legend is extremely vague, i.e. "the toxin induced a behavioral response".  It seems to me that in both cases, an experimental comparison to what is observed upon injection of SNX-482 would be quite essential, and a much more detailed discussion of all of these matters. The n values in these experiments are also much too low (3 and 2).    Along these lines, one of the most prominent roles of R-type channels is their contribution to nociceptive signaling and their role in seizure disorders, including kainate induced seizures.  It seems to me that the authors could have tested the effect of the toxin in a mouse pain or seizure model.  Alternatively, maybe the authors could simply leave out the in vivo experiments and focus in more detail on the target interaction with Cav2.3 as per my first point above.

Answer:

We conducted the physiological studies in mice and zebrafish at an early stage of this work and were pleased to see effects in both models, which convinced us of the bioactivity of Mu8.1. However, as pointed out by the reviewer we do not currently understand the observed effects in mechanistic detail. We have therefore decided to take the advice of the reviewer to leave out the physiological studies of the present work.

*Reviewer comment:*

Supplementary Figure 11:  No quantification or n values are provided for these data unlike in Figure S10.

Answer:

We have now added a quantification and n values to the former Fig. S11 (Fig. S13 in the revised manuscript).

**Reviewer #2**

*Reviewer comment:*

*This manuscript reports the discovery and initial characterization of a novel class of conotoxins, Mu8.1. In contrast to most conotoxins, Mu8.1 is large (89 residues). The authors provide extensive biochemical, structural, and functional analysis. The structural data show that Mu8.1 has a saposin fold and has a reasonable propensity to form dimers. There are clear functional effects in both zebrafish and mouse models that provide evidence that the toxin is bioactive. Profiling shows that Mu8.1 has the highest potency against the R-type calcium channel CaV2.3 (a channel isoform that has rather poor pharmacology). The IC50 for this channel is not particularly high (~5 µM) and not terribly separated from its effects on other voltage-gated channels (~30 µM for a number of related CaVs, as well as a voltage-gated potassium channel). This 'preferential' activity that is mentioned in the abstract needs to be made a bit clearer as without a careful read of the*

*manuscript, readers might get the impression that Mu8.1 is selective for CaV2.3.  Nevertheless, overall the manuscript presents an important advance. There are a number of issues with the clarity of the presentation that should be addressed as*

Answer:

We thank the reviewer for the positive assessment of our manuscript, along with constructive feedback.

As per the reviewer’s suggestion the term ”preferential” has been removed. The revised Abstract now states:

” When tested on a variety of recombinantly expressed voltage-gated ion channels, Mu8.1 displayed the highest potency against the R-type (Cav2.3) calcium channel.”

Furthermore, we explicitly acknowledge in the revised manuscript that although Mu8.1 exhibits the highest potency among mammalian targets towards Cav2.3, the IC_50_ observed against this target is modest (e.g., in line 459 – Source document with track-changes). Additionally, we propose the possibility of conducting mutational screening of Mu8.1 to enhance its potency against Cav2.3 (lines 577-579 – Source document with track-changes).

*Reviewer comment:*

*Abstract last sentence. It is not clear what 'feasibility of large, disulfide rich venom component investigation' means.  Maybe the authors are referring to the fact that they are able to make a bacterial system that can express the toxin? Although this is an important technical step, it probably also could be done by secreting the protein from insect or HEK cells.  It seems like a strange ending sentence for the manuscript as I do not think this is the most important finding of the study.*

Answer:

We have reworded the last sentence of the Abstract resulting in the removal of the confusing wording.

*Reviewer comment:*

*Line 23 'asymmetric, heteromeric'.  By definition, if a K channel is heteromeric it will be asymmetric.*

Answer:

We have deleted the word “asymmetric”.

*Reviewer comment:*

*Lines 130-135  The discussion on the similarity (or not) to the con-ikot-ikot toxins is confusing. First, it seems that the authors are trying to make the comparison based on the signal sequences, which is odd as what matters is whether the toxins themselves have similar folds in the part that is actually active.  This is what the authors eventually get to in the MS, so what starts as a 'misannotation' is actually something that was correct. Since both Mu81. And the con-ikot-ikot toxins share the saponin fold, it would seem that this fact should be described in a less round-about way.  The same fold appears to be used in different ways to engage different targets (glutamate receptors vs. CaV2.3 and relatives). The latter seems like a key point that is undersold here.*

Answer:

We appreciate the reviewer’s suggestion and in the revised manuscript we have removed the confusing wording present in lines 130-135 of the original manuscript. The discussion about the similarity between Mu8.1 and con-ikot-ikot is now described in the paragraph starting in line 164.

We thank the reviewer for the valuable advice regarding the need to clarify that the same fold is employed to interact with multiple targets. We have incorporated this point explicitly into the Discussion section (lines 555-557 – Source document with track-changes).

*Reviewer comment:*

*Line 404  Disulfide bonds are crosslinks. They are not metaphorical crosslinks and should not be in quotation marks.*

Answer:

We have removed the quotation marks around the word crosslinks.

**Reviewer #3**

*Reviewer comment:*

*The present manuscript reports interesting data concerning the identification of the sequence of an unusually large conotoxin, Mu8.1, that defines a new class of conotoxin that inhibits with a preferential selectivity the R-type (Cav2.3) calcium channel. Functional studies using defined classes of murine somatosensory dorsal root ganglion (DRG) neurons show that Mu8.1 effectively blocks the calcium influx across the neurons. When tested on a variety of voltage-gated calcium channels heterologously expressed in HEK 293 cells, Mu8.1 inhibited the Cav2.3) channel with an IC50 of about 5 microM, i.e, only 5 times lower than the other Cav channels (Cav2.1, Cav2.2 and Cav3), see Table S4. Although not impressively effective these new findings highlight the potential of Mu8.1 as a molecular tool to identify and study neuronal subclasses expressing Cav2.3.*

*Reviewer comment:*

*Electrophysiological experiments are on sound but the number "n" should be increases to 4-5 (see Table S4). I do not have major criticisms except suggesting the authors to smooth the emphasis on the potency of the new toxin as a Cav2.3 channel blocker. SNX-482 is far more potent than Mu8.1 and would be hardly replaced in pharmacological studies when assaying Cav channel availability in excitable cells. Nevertheless Mu8.1 represents a good new start concerning the underexplored group of macro-conotoxins that may lead to the discovery of new molecules with high affinity for Cav channels (highly required!).*

Answer:

We thank the reviewer for the positive assessment of our manuscript. We have now increased the number “n” to 5 for all experiments summarized in Table S4.

We appreciate the reviewer's suggestion to provide a more balanced discussion regarding the potency of Mu8.1 as a Cav2.3 channel inhibitor. In the revised manuscript, we have omitted the term "preferential". Furthermore, we explicitly acknowledge that although Mu8.1 exhibits the highest potency among mammalian targets towards Cav2.3, the IC_50_ observed against this target is modest (e.g., in line 459 – Source document with track-changes). Additionally, we propose the possibility of conducting mutational screening of Mu8.1 to enhance its potency against Cav2.3 (lines 577-579 – Source document with track-changes).
